# Integrative and Complementary Health Practices for Chronic Pain: summary of clinical guideline recommendations

**DOI:** 10.1590/S2237-96222025v34e20240771.en

**Published:** 2025-08-11

**Authors:** Marcus Tolentino Silva, Daniel Miele Amado, Paulo Roberto Sousa Rocha, Jorge Otávio Maia Barreto

**Affiliations:** 1 Universidade de Brasília, Departamento de Saúde Coletiva, Brasília, DF, Brazil; 2 Ministério da Saúde, Núcleo Técnico de Gestão da Política Nacional de PICS no SUS, Brasília, DF, Brazil; 3 Fundação Oswaldo Cruz, Gerência Regional de Brasília, Brasília, DF, Brazil

**Keywords:** Clinical Practice Guide, GRADE Approach, Complementary Therapies, Chronic Pain, Evidence-Informed Policy, Guía de Práctica Clínica, Enfoque GRADE, Terapias Complementarias, Dolor Crónico, Política Basada en Evidencia

## Abstract

**Objective:**

To evaluate the recommendations of clinical guidelines that used the Grading of Recommendations Assessment, Development and Evaluation system (GRADE) for Integrative and Complementary Health Practices in the management of chronic pain in adults.

**Methods:**

A summary of evidence based on rapid review, in an agile, rigorous, and timely manner, to inform health decision-making. Searches were conducted in Medline, Embase and Scopus, and included clinical guidelines that assessed the quality of evidence and the strength of recommendations. The methodological quality of the guidelines included was assessed using the Appraisal of Guidelines for Research & Evaluation II tool, aiming to characterize how well-founded and transparent the included guidelines were.

**Results:**

Eighteen guidelines published between 2011 and 2024 were included, eight of which were of high methodological quality. The most recommended practices were acupuncture, osteopathy, chiropractic, yoga, tai chi, meditation, and relaxation techniques, for conditions such as low back pain, chronic pelvic pain, fibromyalgia, irritable bowel syndrome, chronic pancreatitis and rheumatoid arthritis. The quality of the evidence supporting these recommendations was predominantly low to moderate, with recommendations overwhelmingly weak. The interventions were considered effective and had a favorable safety profile compared to conventional treatments. Strong recommendations included acupuncture, osteopathy, chiropractic, and yoga for low back pain.

**Conclusion:**

The quality of the evidence was limited, suggesting the need for further robust studies of interventions for chronic pain management. Acupuncture, osteopathy, chiropractic and yoga presented better evidence, with potential for effectiveness and safety for the Brazilian Unified Health System.

## Resumen

**Objetivo:**

Evaluar las recomendaciones de las guías clínicas que utilizaron el sistema de Grading of Recommendations Assessment, Development and Evaluation para Prácticas de Salud Integrativas y Complementarias en el manejo del dolor crónico en adultos.

**Métodos:**

Síntesis de evidencia basada en revisión rápida, de forma ágil, rigurosa y oportuna, para informar la toma de decisiones en salud. Se realizaron búsquedas en Medline, Embase y Scopus, e incluyeron guías clínicas que evaluaron la calidad de la evidencia y la fuerza de las recomendaciones. La calidad metodológica de las guías incluidas se evaluó utilizando la herramienta Appraisal of Guidelines for Research & Evaluation II, con el objetivo de caracterizar cuán bien fundadas y transparentes eran las guías incluidas.

**Resultados:**

Se incluyeron 18 guías publicadas entre 2011 y 2024, ocho de las cuales fueron de alta calidad metodológica. Las prácticas más recomendadas fueron la acupuntura, la osteopatía, la quiropráctica, el yoga, el tai chi, la meditación y las técnicas de relajación, para afecciones como el dolor lumbar, el dolor pélvico crónico, la fibromialgia, el síndrome del intestino irritable, la pancreatitis crónica y la artritis reumatoide. La calidad de la evidencia que respalda estas recomendaciones fue predominantemente baja a moderada, y las recomendaciones fueron abrumadoramente débiles. Las intervenciones se consideraron efectivas y tuvieron un perfil de seguridad favorable en comparación con los tratamientos convencionales. Entre las recomendaciones más importantes se incluyen la acupuntura, la osteopatía, la quiropráctica y el yoga para el dolor lumbar.

**Conclusión:**

La calidad de la evidencia fue limitada, lo que sugiere la necesidad de realizar más estudios sólidos sobre intervenciones para el manejo del dolor crónico. La acupuntura, la osteopatía, la quiropráctica y el yoga presentaron mejor evidencia, con potencial de efectividad y seguridad para el Sistema Único de Salud.

**Ethical aspects**

This research used public domain data and anonymized databases.

## Introduction

Chronic pain represents a major challenge for global public health, impacting the quality of life of millions of people and generating high costs for health systems, including the Brazilian Unified Health System (SUS) (1,2). This condition affects people around the world, and is generally associated with increased medical costs, work absenteeism and reduced quality of life, in addition to other issues related to the different approaches adopted to address it within health systems and services (3,4). Unlike acute pain, which has a protective biological function, chronic pain can persist for months or years, becoming a complex condition that requires multidisciplinary approaches (5). In the context of health policies, its management requires access to conventional treatments and integrated strategies that consider biopsychosocial aspects. Overburdened health systems struggle to provide comprehensive and accessible care, especially in the face of overreliance on painkillers and opioids, which can be accompanied by increased risks and adverse effects (6,7).

In Brazil, the National Policy for Integrative and Complementary Practices in the SUS (PNPIC) was established in 2006 and updated in 2017 and 2018 (8). The PNPIC has pointed to the use of different therapies, such as acupuncture, meditation, chiropractic and herbal medicine, as options for the management of chronic pain, also reinforcing the relevance of research on Integrative and Complementary Practices (ICPs). In general, many of these practices are already recognized by the World Health Organization (WHO) and have demonstrated benefits for the well-being of patients (9). Despite these regulatory advances, implementation challenges persist for well-informed judgment on effectiveness and sustainability in the SUS and other health systems, such as gaps in clinical practice recommendations, professional training initiatives, expansion of the supply in public services and scientific basis.

In this context, ICPs are attracting a growing interest among patients and professionals (10). From the perspective of health services, the decision to use ICPs is a complex and dynamic process, influenced by personal, social and contextual factors (11,12). From diagnosis and throughout the evolution of their disease, patients seek information about ICPs from different sources, weighing beliefs, values and the credibility of the available evidence (13). Decision-making is very personal: some patients prioritize the relationship with the health professional, the severity of the disease and the willingness to try innovative approaches, while others value intuition or seek robust evidence of efficacy. Factors such as trust in the professional, previous experiences and the influence of social networks also play an important role (12). Decisions about the use of ICPs are often reassessed at key moments, such as the completion of conventional treatment or the emergence of new information (11).

On the other hand, the recommendations contained in clinical guidelines and therapeutic protocols are essential for the effectiveness and sustainability of health systems, as they guide conduct based on the best available evidence, in a contextualized manner, to ensure more effective and safe care, reduce unnecessary clinical variations, and optimize resources. They improve the quality of care, avoid waste and contain costs by preventing ineffective treatments and complications that overload the system (14). Thus, well-structured guidelines can help achieve a balance between quality, access and financial viability, making health systems more efficient and sustainable in the long term.

Faced with this problem, it is strategic to perform a critical and systematic analysis of the best available evidence. This analysis must use rigorous methods to support decisions in public health policies. This includes the development of national clinical guidelines that ensure safe and effective practices for SUS. In the context of developing recommendations for clinical practice, the Grading of Recommendations Assessment, Development and Evaluation system (GRADE) is a structured and transparent tool for assessing the quality of evidence and the strength of recommendations in clinical guidelines. It allows a careful analysis of studies, considering risk of bias, inconsistencies, applicability and clinical impact, differentiating strong from weak recommendations. GRADE also integrates factors such as benefits, risks, patient preferences and cost-effectiveness, facilitating informed decisions. Its standardization improves transparency in scientific communication and contributes to more consistent, adaptable and sustainable guidelines, ensuring that clinical practice is guided by the best available knowledge and promoting efficient use of health resources (15).

The GRADE system is widely used by health organizations around the world, including the WHO and several national agencies, to develop guidelines based on robust and well-evaluated evidence. Mapping clinical guidelines adopted in other countries and settings can serve as a reference to allow health systems to advance in the development of their own recommendations, adapting them to their epidemiological, social and economic context (16). This would avoid the need to start the entire process over again, accelerating the implementation of good practices and enabling more efficient use of resources, as decisions are informed by the best evidence available globally. Thus, the question of this research was: what are the main recommendations of GRADE clinical guidelines for the use of ICPs in the treatment of chronic pain in adults?

In this sense, the present study aims to map the recommendations of clinical guidelines that use the GRADE system for the use of ICPs in the treatment of chronic pain in adults.

## Methods

This is a summary of evidence, based on a rapid review method for health technology analysis (17). This method uses methodological shortcuts to synthesize the best available evidence in a timely manner, without compromising the quality and reliability of the results. In this review, these shortcuts include delimiting the scope of the question, prioritizing published secondary studies (clinical guidelines), using specific tools for critical appraisal, and simplifying the data extraction process, which was conducted in a non-duplicated manner.

### Eligibility criteria

The eligibility criteria for inclusion of studies in this summary of evidence were defined as follows: clinical guidelines published in scientific journals that used the GRADE system to extract the quality of evidence and the strength of recommendations, which addressed the use of ICPs for the treatment of chronic pain in adult patients, available in any language, on any date of publication. The choice of GRADE guidelines is justified by their robust and transparent method. The inclusion of guidelines regardless of language and publication date aimed to broaden the scope of the summary and the representativeness of the recommendations. The decision to include only published guidelines in the search is justified by the need to guarantee the quality, credibility and methodological rigor of the evidence analyzed. The exclusion of gray literature, such as preprints and non-reviewed institutional documents, aims to maintain the focus on robust evidence that is widely accepted by the scientific community, strengthening the validity of this summary of evidence.

### Sources of information

The information sources used to search for clinical guidelines were Medline (via PubMed), Embase and Scopus, recognized for indexing scientific journals in health and biomedical sciences. The search of these sources aimed to increase the likelihood of finding eligible guidelines.

### Search strategy

The search strategy was developed by the authors in all its phases, including preparation, preliminary testing and application. Table 1 describes the details of the search strategy used in this study, with a focus on identifying published clinical guidelines that addressed the treatment of chronic pain. The strategy combines terms related to “chronic pain”, “guidelines” and “GRADE system”. The search was refined by the authors for each database, using Boolean operators and terms specific to each platform, as shown in Table 1, to maximize sensitivity and specificity.

**Table 1:** Search strategy in selected information sources

Source	Search terms	Recovered studies
Medline (via PubMed)	#1 “chronic pain”[Mesh] OR (chronic pain) #2 (guidelines as topic[MeSH:noexp] OR practice guidelines as topic[MeSH:noexp] OR Health Planning Guidelines[MeSH:noexp] OR practice guideline[MeSH:noexp] OR clinical protocols[MeSH:noexp] OR Consensus[MeSH:noexp] OR “Consensus Development Conference”[PTYP] OR “Consensus Development Conference, NIH”[PTYP] OR “Consensus Development Conferences as Topic”[MeSH:noexp] OR “Consensus Development Conferences, NIH as Topic”[MeSH:noexp] OR critical pathway[MeSH:noexp] OR (clinical[TIAB] AND pathway[TIAB]) OR (clinical[TIAB] AND pathways[TIAB]) OR (practice[TIAB] AND parameter[TIAB]) OR (practice[TIAB] AND parameters[TIAB]) OR algorithms[MeSH:noexp] OR care pathway[TIAB] OR care pathways[TIAB] OR guidance[TIAB] OR guideline*[TI]) #3 “GRADE system”[tiab] OR “GRADE methods”[tiab] OR “GRADE approaches”[tiab] OR “GRADE approach”[tiab] OR “Grading of Recommendations, Assessment, Development and Evaluation”[tiab] OR (“GRADE”[tiab] AND (“low”[tiab] OR “moderate”[tiab]OR “high”[tiab] OR “evidence”[tiab] OR “strong”[tiab] OR “weak”[tiab] “recommendation”[tiab])) #4 #1 AND #2 AND #3	96
Embase	#1 ‘chronic pain’/exp OR ‘chronic pain’ #2 ‘practice guideline’/exp OR ‘practice guideline’ #3 ‘GRADE approach’/exp OR ‘GRADE approach’ #4 #1 AND #2 AND #3	30
Scopus	(TITLE-ABS-KEY (“chronic pain”)) AND (TITLE-ABS-KEY(guidelines)) AND (TITLE-ABS-KEY(“GRADE system” OR “GRADE methods” OR “GRADE approaches” OR “GRADE approach” OR “Grading of Recommendations, Assessment, Development and Evaluation”))	120

### Selection process

The selection of guidelines was made in two stages, with the help of an electronic spreadsheet. First, two independent reviewers screened the titles and abstracts of the records identified in the databases, using the predefined eligibility criteria. Disagreements between reviewers were resolved by consensus. Records considered potentially eligible after initial screening then had their full text retrieved for detailed assessment by one reviewer, with eligibility assessed by another reviewer. A PRISMA flow diagram was developed to illustrate the process.

### Data collection process

One reviewer extracted data from the included guidelines using a standardized spreadsheet and a second reviewer reviewed the extracted information for confirmation purposes. Disagreements in data extraction were discussed by the reviewers, reaching a consensus.

### Data list

A spreadsheet was developed to collect the following data: year of publication; place of origin; target population (type of chronic pain); ICPs included; outcomes considered; recommendation for the use of ICPs; quality of evidence (high, moderate, low, or very low) and strength of recommendation (strong or weak).

### Assessment of risk of bias in studies

To assess the risk of bias of the clinical guidelines included in the summary, the methodological rigor domain of the Appraisal of Guidelines for Research and Evaluation II (AGREE II) tool was used (18). The following items were used: (i) Were systematic methods used to search for evidence? (ii) Are the criteria for selecting evidence clearly described? (iii) Are the strengths and limitations of the body of evidence clearly described? (iv) Are the methods for formulating the recommendations clearly described? (v) Have the benefits, side effects, and health risks been considered in formulating the recommendations? (vi) Is there an explicit relationship between the recommendations and the evidence supporting them? (vii) Has the guideline been externally reviewed by experts before its publication? (viii) Is a procedure for updating the guideline available? The assessment was applied by one reviewer and checked by another. Any disagreements were resolved by consensus. Both reviewers had previous experience in applying AGREE II.

### Summary methods

The analysis of the recommendations of the guidelines included in the summary of evidence was conducted in a narrative manner, considering the methodological quality of the guidelines and the quality of the evidence supporting each recommendation. The recommendations were grouped by the type of chronic pain and modality of ICPs, seeking to identify points of convergence and divergence between the different guidelines.

### Certainty assessment

The analysis of the results considered the methodological quality of the guidelines, giving greater weight to recommendations from guidelines with a minimal risk of bias, i.e., the most dependable. Guidelines with high confidence were considered those with the highest number of “yes” responses in the methodological rigor of the AGREE II tool; guidelines with moderate confidence were those with a balance of “yes”, “partially”, and “no” responses; and guidelines with low confidence were those with the majority of criteria classified as “partially” and “no”.

The strength of recommendations and quality of evidence were extracted from the guidelines’ GRADE system. In GRADE, a strong recommendation indicates that the expert panel is confident that the beneficial effects outweigh the harm. A weak/conditional recommendation means that there is uncertainty about the balance of benefits and risks, so the best decision may vary depending on the context or the patient’s values. The combination of recommendations was conducted in a qualitative manner, seeking a balance based on a critical analysis of the set of available evidence.

## Results

**Figure 1:**
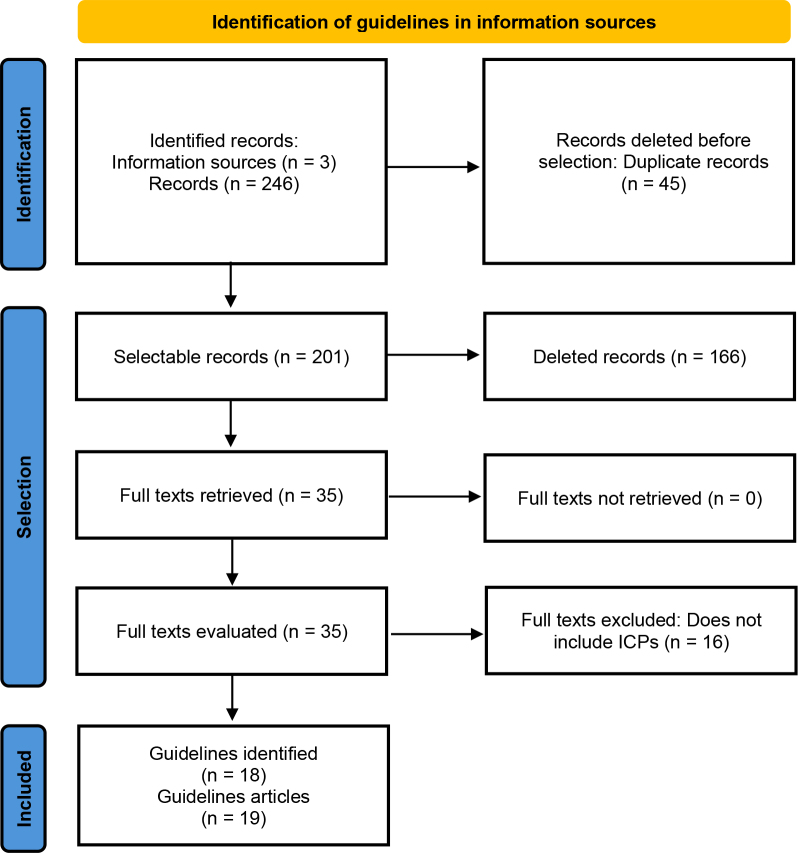
Flowchart for identifying guidelines on chronic pain and the use of Integrative and Complementary Health Practices

Figure 1 presents the process of selecting guidelines for the summary of evidence. The search for information sources on September 9, 2024, resulted in 246 records. After removing duplicates, 201 records were considered for the selection of titles and abstracts. Of these, 35 had their full text retrieved for evaluation. After reading the full texts, 19 articles describing 18 guidelines were included in the summary because they addressed some type of chronic pain and had some ICPs among their recommendations (19-37).

Table 2 describes the characteristics of the 18 clinical guidelines included in this summary published between 2011 and 2024. The guidelines come from multiple countries, with emphasis on European countries and the United States. Target populations included conditions such as low back pain, chronic pelvic pain, fibromyalgia, irritable bowel syndrome, chronic pancreatitis, and rheumatoid arthritis. The most recommended ICPs were acupuncture, osteopathy, chiropractic, yoga, tai chi, meditation and relaxation techniques. The outcomes assessed included reduction in pain intensity, improvement in physical and emotional functionality, quality of life, sleep, psychological well-being and reduction in medication use.

**Table 2:** Characterization of included clinical guidelines on chronic pain

Author/year	Place of origin	Target population	Integrative and Complementary Practices in Health	Outcomes
Allaire 2024 (19)	Canada	Adolescent women with chronic pelvic pain	Acupuncture	Pain reduction
Brandow 2020 (20)	United States	Children and adults with sickle cell disease	Acupuncture; therapeutic massage	Pain relief, reduced use of painkillers, psychological well-being.
Broder 2022 (21)	United States	Adults with low-risk recurrent abdominal pain presenting to the emergency room	Acupuncture	Reduction in pain, need for rescue analgesics, and patient satisfaction with pain relief.
Busse 2023 (22)	Multiple countries	Adult patients with chronic pain (≥3 months) associated with temporomandibular disorders	Acupuncture	Pain relief, physical functioning, emotional functioning, role functioning, social functioning, sleep quality, and adverse events.
Coulter 2019 (23)	United States	Patients with unspecified neck pain	Osteopathy; chiropractic	Reduced pain, reduced disability, and improved quality of life.
From Andres 2016 (24)	Spain	Women with vulvodynia	Biofeedback; cognitive behavioral therapy	Reduction of pain, dyspareunia, improvement of quality of life.
Di Nardo 2024 (25)	Italy	Children and adolescents with irritable bowel syndrome	Yoga; Benson relaxation technique	Reduction in the frequency and intensity of abdominal pain, improvement in quality of life, reduction in the severity of symptoms and psychological well-being.
Dominguez-Munoz 2018 (26)	Europe	Patients with chronic pancreatitis	Acupuncture	Pain relief, improvement in quality of life, nutritional status, diabetes control, disease progression and complications.
Hawk 2020 (27)	United States	Patients with chronic musculoskeletal pain	Acupuncture; osteopathy; chiropractic; yoga; meditation mindfulness; cognitive behavioral therapy	Pain control, function, quality of life, mental health, pain-related behaviors, medication use, patient satisfaction, costs, and adverse events.
Krenn 2020 (28)	Austria	Adults with nonspecific low back pain	Acupuncture; traditional Chinese medicine; herbal medicine; osteopathy; chiropractic; yoga; meditation; music therapy	Pain reduction, improved functionality, reduced medication use.
Larun 2011 (29)	Norway	Patients with chronic fatigue syndrome	Qigong	Levels of fatigue, pain, and health-related quality of life.
Lohr 2017 (30)	Europe	Patients with chronic pancreatitis	Acupuncture	Pain relief, quality of life, morbidity, mortality, and treatment costs.
Moisset 2020 (31, 32)	France	Patients with chronic neuropathic pain, including adults and children	Ear acupuncture; aromatherapy; aromatherapy massage; low intensity laser	Pain intensity, neuropathic symptoms and signs, sleep, quality of life, comorbidities, clinical global impression of change and adverse events.
Pangarkar 2019 (33)	United States	Patients with low back pain (including war veterans and military personnel)	Acupuncture; osteopathy; chiropractic; yoga; meditation mindfulness; cognitive behavioral therapy	Pain, function, quality of life, mental health, pain-related behaviors, medication use, patient satisfaction, costs, and adverse events.
Qaseem 2017 (34)	United States	Adults with acute, subacute, or chronic low back pain	Acupuncture; osteopathy; chiropractic; massage; tai chi; yoga; mindfulness	Pain reduction, function improvement, health-related quality of life improvement, work disability reduction, return to work, overall improvement, number of low back pain episodes or time between episodes, patient satisfaction.
Sauvan 2018 (35)	France	Women with endometriosis without infertility	Yoga; osteopathy; acupuncture	Improved quality of life and reduced pain.
Van Doorn 2023 (36)	Europe	Patients with Guillain-Barré syndrome	Yoga; meditation	Sleep quality, pain, daily activities, and quality of life.
Zhang 2024 (37)	Multiple countries	People with active rheumatoid arthritis	Extract from *Tripterygium wilfordii* *Hook F*	Clinical response (ACR20/50/70^a^, DAS28^b^, EULAR^c^, cDAI^d^), patient-rated pain (VAS^e^), quality of life (SF-36^f^, HAQ^g^), radiographic progression, safety, and cost.

^a^ American College of Rheumatology Response Criteria 20%, 50% and 70% improvement; ^b^ Disease Activity Score in 28 Joints; ^c^ European League Against Rheumatism; ^d^ Clinical Disease Activity Index; ^e^ Visual Analog Scale; ^f^ Short Form 36; ^g^ Health Assessment Questionnaire.

Table 3 presents an assessment of the methodological quality of the clinical guidelines included in the summary. Eight guidelines demonstrated high methodological quality, indicating greater methodological rigor and transparency in the development process. The other guidelines presented flaws in criteria such as the detailed description of the methods for searching and selecting evidence, the critical analysis of the body of evidence and the explanation of the relationship between the recommendations and the evidence, resulting in moderate or low-quality classifications.

**Table 3:** Critical evaluation of included clinical guidelines on chronic pain

Author/year	1^a^	2^b^	3^c^	4^d^	5^e^	6^f^	7^g^	8^h^	Guideline confidence
Allaire 2024 (19)	S^i^	P^j^	S	S	S	S	S	S	Moderate
Brandow 2020 (20)	S	P	S	S	S	P	S	S	Moderate
Broder 2022 (21)	S	S	S	S	S	P	S	S	Moderate
Busse 2023 (22)	S	S	S	S	S	S	N^l^	N	Moderate
Coulter 2019 (23)	S	S	P	N	S	S	S	N	Low
From Andres 2016 (24)	S	P	S	N	P	S	N	N	Low
Di Nardo 2024 (25)	S	S	S	S	S	S	S	N	High
Dominguez-Munoz 2018 (26)	P	N	P	P	S	P	S	N	Low
Hawk 2020 (27)	S	S	S	S	S	S	S	N	High
Krenn, 2020 (28)	S	S	S	N	P	S	N	P	Low
Larun 2011 (29)	S	S	S	N	S	P	N	N	Low
Lohr 2017 (30)	S	S	S	S	S	S	S	S	High
Moisset 2020 (31, 32)	S	S	S	S	S	S	S	N	High
Pangarkar 2019 (33)	S	S	S	S	S	S	S	N	High
Qaseem 2017 (34)	S	S	S	S	S	S	S	P	High
Sauvan 2018 (35)	S	P	S	S	S	S	S	N	Moderate
Van Doorn 2023 (36)	S	S	S	S	S	S	S	S	High
Zhang 2024 (37)	S	S	S	S	S	S	S	N	High

^a^1. Were systematic methods used to search for evidence? ^b^ 2. Are the criteria for selecting evidence clearly described? ^c^ 3. Are the strengths and limitations of the body of evidence clearly described? ^d^ 4. Are the methods for formulating recommendations clearly described? ^e^ 5. Were the benefits, side effects and health risks considered in formulating the recommendations? ^f^ 6. Is there an explicit relationship between the recommendations and the evidence that supports them? ^g^ 7. The guideline was externally reviewed by experts before its publication?; ^h^ 8. Is a procedure for updating the guideline available? ^i^ Yes; ^j^ Partially; ^l^ No.

Table 4 summarizes the recommendations for the use of ICPs for distinct types of chronic pain. Recommendations vary depending on the specific condition and ICPs modality. Most guidelines suggest its use as a complementary treatment, emphasizing the importance of considering patient preferences. The quality of the evidence supporting these recommendations is predominantly low to moderate, resulting in mostly weak recommendations. Confidence in the guidelines also varies, from low to high. The guidelines with the highest confidence recommend some ICPs for chronic pain management with reservations. Acupuncture, osteopathy, chiropractic, yoga and tai chi have strong recommendations, suggesting greater support for their efficacy for low back pain. It is noteworthy that, even in the most confident guidelines, the recommendations are weak, reflecting the limitations of the available evidence.

**Table 4:** Summary of recommendations for the use of Integrative and Complementary Health Practices in clinical guidelines on chronic pain

Target population	Recommendation	Quality of evidence	Strength of recommendation	Guideline confidence
**Musculoskeletal system**				
Temporomandibular disorders	Acupuncture may be considered (22).	Low	Weak	Moderate
Neck pain	Osteopathy and chiropractic may be considered viable options for pain management and disability reduction (23).	Moderate	Weak	Low
	Acupuncture may be beneficial as part of a multimodal treatment plan, especially early on, to help the patient become or remain active (27).	Moderate	Weak	High
Acute or subacute low back pain	Acupuncture, osteopathy and chiropractic may be considered (34).	Low	Strong	High
Chronic low back pain	Acupuncture may be offered (28,33,34). Yoga and tai chi can be offered as part of an exercise program (28,33,34).	Low	Strong	High
Chronic pelvic pain	Massage can be considered (34).	Moderate	Weak	High
	Acupuncture can be considered a complementary modality (19,35).	Low	Weak	Moderate
Fibromyalgia	Acupuncture can be beneficial (27).	Moderate	Weak	High
Osteoarthritis	Acupuncture, using “high dose” (higher frequency of treatment, at least three times per week), may be beneficial (27).	Moderate	Weak	High
**Systemic and inflammatory conditions**				
Rheumatoid arthritis	Monotherapy with extract of *Tripterygium wilfordii Hook F* or its association with methotrexate may be a first-line option (37).	Moderate	Weak	High
Sickle cell disease	Therapeutic massage and acupuncture should be suggested as available, tolerated and conditioned on individual patient preference and response (20). These approaches should be provided within the context of a comprehensive disease and pain management plan. (20).	Very low	Weak	Moderate
Chronic neuropathic pain	Cognitive-behavioral therapy and mindfulness may be recommended as second-line therapy in addition to other therapies (31, 32). Hypnosis, reflexology, and acupuncture may be effective, but further research is needed (31, 32).	Moderate	Weak	High
Endometriosis	Yoga, osteopathy and acupuncture can be proposed as part of the non-drug approach to improve quality of life (35).	Low	Weak	Moderate
Chronic pancreatitis	Acupuncture may be considered in cases of pain (26,30).	Moderate	Weak	High
Irritable bowel syndrome	Yoga and relaxation can be considered (25).	Very low	Weak	High
Chronic fatigue syndrome	Therapeutic exercise may be recommended, as long as the program is adapted to the nature of the disease (29).	Moderate	Weak	Low
Guillain-Barré syndrome	Yoga and meditation may be considered for treating pain and fatigue, but more research is needed (36).	Very low	Weak	High
**Other conditions and symptoms**				
Abdominal pain	Acupuncture may be considered (21).	Very low	Weak	Moderate
Vulvodynia	Biofeedback can be considered as part of a multidisciplinary approach, especially in cases of pelvic floor dysfunction (24). Cognitive behavioral therapy is recommended as part of a multidisciplinary approach, along with supportive psychotherapy (24).	Moderate	Weak	Low

## Discussion

Clinical guidelines that contemplate the use of ICPs for chronic pain reveal a scenario of growing interest, but with a still limited evidence base. Although many guidelines, especially European and American, address the topic for different painful conditions, the methodological quality of these guidelines is heterogeneous, with many presenting weaknesses in the transparency and rigor of the development process. Except for the use of acupuncture, osteopathy, chiropractic, yoga and tai chi for low back pain, the recommendations are weak, reflecting predominantly low to moderate quality evidence. This suggests that, although there is evidence of the potential benefit of ICPs in the management of chronic pain, more robust research is needed to consolidate these practices and define their role more precisely in clinical practice. The emphasis on individualizing treatment and considering patient preferences reinforces the need for a cautious and integrated approach, based on evidence and shared communication.

The summary of evidence has limitations. The heterogeneity of the clinical guidelines included, both in terms of scope (different types of chronic pain) and methodological quality, makes it difficult to generalize the conclusions (38). The predominance of low- to moderate-quality evidence supporting the recommendations introduces imprecision and limits the strength of the conclusions (39). Potential publication bias, with negative or inconclusive studies being less likely to be published, may have influenced the results (40). The search for guidelines, even when conducted in relevant databases, did not capture all existing guidelines, especially those not indexed. 

ICPs have potential for the management of chronic pain, offering a cost-effective option with a favorable safety profile compared to conventional treatments. Modalities such as acupuncture and mind-body practices address the complexity of chronic pain while minimizing the adverse effects often associated with medications (41). Careful integration of ICPs into conventional medicine can optimize resources, reduce side effects, and increase patient satisfaction (42). This summary points out the most promising ICPs for each type of chronic pain, which can be integrated with conventional treatments available in the SUS (Brazilian Unified Health System).

Acupuncture, for example, is already included in the National Policy for Integrative and Complementary Practices in the SUS, and has demonstrated benefits in international guidelines, representing an opportunity to expand access and reduce inequities (43). Training SUS professionals in ICPs, such as yoga and meditation, with the potential to be offered at distinct levels of care, can strengthen patient autonomy and health promotion. The careful inclusion of ICPs expands therapeutic options, promotes comprehensive care, and strengthens the SUS, ensuring equitable access to safe and effective practices.

The Municipal Health Department of São Paulo standardized the management of chronic pain in reference centers in the city (44). Although the focus is on drug treatment and conventional interventions, the importance of ICPs is recognized as part of a multidisciplinary approach. Among the ICPs mentioned are acupuncture, auriculotherapy, moxibustion, reflexology, tai chi, cupping therapy, herbal medicine, meditation, and yoga. The clinical protocol cites systematic reviews that demonstrate the effectiveness of these practices for several types of chronic pain, including low back, neck, musculoskeletal, and cancer pain. The inclusion of ICPs in the protocol reinforces the trend of integrating these approaches into the SUS, aiming at a more comprehensive and individualized management of chronic pain.

The Clinical Protocol and Therapeutic Guidelines for Chronic Pain (PCDT), published in August 2024, recognizes the importance of ICPs. Among the ICPs highlighted are acupuncture and auriculotherapy, which have shown effectiveness in reducing pain in adults and the elderly (45). These practices are recommended as part of a multidisciplinary therapeutic approach, aiming to improve patients’ quality of life. The PCDT emphasizes the need for professionals trained to apply these techniques, ensuring the safety and effectiveness of the treatment. The importance of considering the preferences and particularities of each patient when preparing the therapeutic plan is also reinforced.

In conclusion, this summary of evidence demonstrates that although the use of PICS for chronic pain is promising and growing, the quality of evidence remains a limiting factor for formulating robust recommendations. The recommendations are strengthened when considering the cost-effectiveness and safety of these practices. Acupuncture, osteopathy, chiropractic, and yoga emerge as practices with the greatest support for low back pain. Healthcare professionals should integrate this evidence with their clinical experience, considering the individual preferences and needs of each patient, when deciding on the use of ICPs. Expanding access to ICPs in the SUS, professional training, and investment in research in the Brazilian context are essential to promote the safe and effective use of these practices, contributing to comprehensive care and the sustainability of the system.

## Data Availability

No database and/or analysis codes were created in this research, other than the illustrations already available in the manuscript.
