# Supplementary material for: Integrative and Complementary Health Practices for Chronic Pain: summary of clinical guideline recommendations
Source: Epidemiol Serv Saude. 2025 Aug 11;34:e20240771. doi: 10.1590/S2237-96222025v34e20240771.en (PMC12342850; doi:10.1590/S2237-96222025v34e20240771.en)
Supplement: Translation

# Translation: Práticas Integrativas e Complementares em Saúde para dor crônica: síntese de recomendações de diretrizes clínicas

**DOI:** 10.1590/S2237-96222025v34e20240771.pt

**Authors:** Marcus Tolentino Silva, Daniel Miele Amado, Paulo Roberto Sousa Rocha, Jorge Otávio Maia Barreto

**Affiliations:** 1 Universidade de Brasília, Departamento de Saúde Coletiva, Brasília, DF, Brasil; 2 Ministério da Saúde, Núcleo Técnico de Gestão da Política Nacional de PICS no SUS, Brasília, DF, Brasil; 3 Fundação Oswaldo Cruz, Gerência Regional de Brasília, Brasília, DF, Brasil

**Keywords:** Guia de Prática Clínica, Abordagem GRADE, Terapias Complementares, Dor Crônica, Política Informada por Evidências

## Abstract

**Objetivo:**

Avaliar as recomendações de diretrizes clínicas que usaram o sistema Grading of Recommendations Assessment, Development and Evaluation para Práticas Integrativas e Complementares em Saúde no manejo da dor crônica em adultos

**Métodos:**

Síntese de evidências baseada em revisão rápida, de forma ágil, rigorosa e oportuna, para informar a tomada de decisão em saúde. As buscas foram feitas nas bases Medline, Embase e Scopus, e incluíram diretrizes clínicas que avaliaram a qualidade da evidência e a força das recomendações. A qualidade metodológica das diretrizes incluídas foi avaliada com a ferramenta Appraisal of Guidelines for Research & Evaluation II, visando à caracterização do quão bem fundamentadas e transparentes foram as diretrizes incluídas.

**Resultados:**

Foram incluídas 18 diretrizes publicadas entre 2011 e 2024, sendo oito com alta qualidade metodológica. As práticas mais recomendadas foram acupuntura, osteopatia, quiropraxia, ioga, tai chi, meditação e técnicas de relaxamento, para condições como dor lombar, dor pélvica crônica, fibromialgia, síndrome do intestino irritável, pancreatite crônica e artrite reumatoide. A qualidade da evidência que sustenta essas recomendações foi predominantemente baixa a moderada, com recomendações majoritariamente fracas. As intervenções foram consideradas efetivas e com perfil de segurança favorável em comparação com tratamentos convencionais. Recomendações fortes incluíram acupuntura, osteopatia, quiropraxia e ioga para dor lombar.

**Conclusão:**

A qualidade das evidências foi limitada, sugerindo a necessidade de mais estudos robustos sobre intervenções no manejo da dor crônica. Acupuntura, osteopatia, quiropraxia e ioga apresentaram melhores evidências, com potencial de eficácia e segurança para o Sistema Único de Saúde.

**Aspectos éticos**

Esta pesquisa utilizou bancos de dados de domínio público e anonimizados.

## Introdução

A dor crônica representa um desafio importante para a saúde pública global, impactando a qualidade de vida de milhões de pessoas e gerando custos elevados para os sistemas de saúde, incluindo o Sistema Único de Saúde (SUS) (1,2). Essa condição afeta populações em todo o mundo, e geralmente está associada à elevação dos custos médicos, absenteísmo laboral e redução da qualidade de vida, além de outras questões relacionadas com as diferentes abordagens adotadas para enfrentá-la no âmbito dos sistemas e serviços de saúde (3,4). Diferentemente da dor aguda, que tem uma função biológica protetora, a dor crônica pode persistir por meses ou anos, tornando-se uma condição complexa que exige abordagens multidisciplinares (5). No contexto das políticas de saúde, seu manejo requer o acesso a tratamentos convencionais e estratégias integradas que considerem aspectos biopsicossociais. Sistemas de saúde sobrecarregados enfrentam dificuldades em oferecer cuidados abrangentes e acessíveis, especialmente diante da dependência excessiva de medicamentos analgésicos e opioides, o que podem ser acompanhado pelo aumento dos riscos e efeitos adversos (6,7).

No Brasil, a Política Nacional de Práticas Integrativas e Complementares no SUS (PNPIC) foi instituída em 2006 e atualizada em 2017 e 2018 (8). A PNPIC tem apontado para o uso de diferentes terapias, como acupuntura, meditação, quiropraxia e fitoterapia, como opções para o manejo da dor crônica, reforçando também a relevância das pesquisas sobre PICS. De forma geral, muitas dessas práticas já são reconhecidas pela Organização Mundial da Saúde (OMS) e têm demonstrado benefícios para o bem-estar dos pacientes (9). Apesar desses avanços normativos, ainda persistem desafios de implementação que permitam o julgamento bem-informado sobre a efetividade e sustentabilidade no SUS e em outros sistemas de saúde, como lacunas nas recomendações de prática clínica, iniciativas de capacitação profissional, ampliação da oferta nos serviços públicos e base científica.

Nesse contexto, as PICS despertam crescente interesse entre pacientes e profissionais (10). Na perspectiva dos serviços de saúde, a decisão de utilizar PICS é um processo complexo e dinâmico, influenciado por fatores pessoais, sociais e contextuais (11,12). Desde o diagnóstico e ao longo da evolução de sua doença, pacientes buscam informações sobre PICS em diversas fontes, ponderando crenças, valores e a credibilidade das evidências disponíveis (13). A tomada de decisão é individualizada: alguns pacientes priorizam a relação com o profissional de saúde, a gravidade da doença e a disposição para experimentar novas abordagens, enquanto outros valorizam a intuição ou buscam evidências robustas de eficácia. Fatores como a confiança no profissional, experiências prévias e a influência de redes sociais também desempenham papel importante (12). Decisões sobre o uso de PICS são frequentemente reavaliadas em momentos-chave, como a conclusão de um tratamento convencional ou o surgimento de novas informações (11).

Noutra vertente, as recomendações contidas nas diretrizes clínicas e protocolos terapêuticos são essenciais para a efetividade e sustentabilidade dos sistemas de saúde, pois orientam condutas a partir das melhores evidências disponíveis, de forma contextualizada, para garantir cuidados mais eficazes e seguros, reduzir variações clínicas desnecessárias e otimizar recursos. Eles melhoram a qualidade assistencial, evitam desperdícios e contêm custos, ao prevenir tratamentos ineficazes e complicações que sobrecarregam o sistema (14). Assim, diretrizes bem estruturadas podem ajudar a alcançar o equilíbrio entre qualidade, acesso e viabilidade financeira, tornando os sistemas de saúde mais eficientes e sustentáveis a longo prazo.

Diante dessa problemática, é estratégico realizar uma análise crítica e sistemática das melhores evidências disponíveis. Essa análise deve utilizar métodos rigorosos para subsidiar decisões nas políticas públicas de saúde. Isso inclui a elaboração de diretrizes clínicas nacionais que garantam práticas seguras e eficazes no SUS. No contexto da elaboração de recomendações para a prática clínica, o sistema Grading of Recommendations Assessment, Development and Evaluation (GRADE) é uma ferramenta estruturada e transparente para avaliar a qualidade das evidências e a força das recomendações em diretrizes clínicas. Ele permite uma análise criteriosa dos estudos, ao considerar risco de viés, inconsistências, aplicabilidade e impacto clínico, diferenciando recomendações fortes de fracas. O GRADE também integra fatores como benefícios, riscos, preferências dos pacientes e custo-efetividade, facilitando decisões embasadas. Sua padronização melhora a transparência na comunicação científica e contribui para diretrizes mais consistentes, adaptáveis e sustentáveis, garantindo que a prática clínica seja guiada pelo melhor conhecimento disponível e promovendo um uso eficiente dos recursos em saúde (15).

O sistema GRADE é amplamente utilizado por organizações de saúde em todo o mundo, incluindo a OMS e diversas agências nacionais, para desenvolver diretrizes baseadas em evidências robustas e bem avaliadas. O mapeamento das diretrizes clínicas adotadas em outros países e cenários pode servir como referência para permitir que os sistemas de saúde avancem na elaboração de suas próprias recomendações, adaptando-as ao seu contexto epidemiológico, social e econômico (16). Isso evitaria a necessidade de recomeçar todo o processo, acelerando a implementação de boas práticas e possibilitando o uso mais eficiente dos recursos, uma vez que as decisões sejam informadas pelas melhores evidências disponíveis globalmente. Assim, a pergunta desta pesquisa foi: quais são as principais recomendações de diretrizes clínicas GRADE, para o uso de PICS no tratamento da dor crônica em adultos?

Nesse sentido, o presente estudo tem como objetivo mapear as recomendações de diretrizes clínicas que utilizam o sistema GRADE para o uso de PICS no tratamento da dor crônica em adultos.

## Métodos

Trata-se de uma síntese de evidências, baseada em método de revisão rápida para avaliações de tecnologias em saúde (17). Este método utiliza atalhos metodológicos para sintetizar as melhores evidências disponíveis em um prazo oportuno, sem comprometer a qualidade e a confiabilidade dos resultados. Nesta revisão, esses atalhos incluem a delimitação do escopo da pergunta, a priorização de estudos secundários publicados (diretrizes clínicas), a utilização de ferramentas específicas para avaliação crítica e a simplificação do processo de extração de dados, que foi realizado de forma não duplicada.

### Critérios de elegibilidade

Os critérios de elegibilidade para a inclusão de estudos nesta síntese de evidências foram assim definidos: diretrizes clínicas publicadas em periódicos científicos que utilizaram o sistema GRADE para extrair a qualidade da evidência e a força das recomendações, que abordassem o uso de PICS para tratamento de dor crônica em pacientes adultos, disponíveis em qualquer idioma, em qualquer data de publicação. A escolha por diretrizes GRADE justifica-se por seu método robusto e transparente. A inclusão de diretrizes independentemente do idioma e da data de publicação visou ampliar o escopo da síntese e a representatividade das recomendações. A decisão de incluir apenas diretrizes publicadas na busca justifica-se pela necessidade de garantir a qualidade, a credibilidade e o rigor metodológico das evidências analisadas. A exclusão de literatura cinzenta, como pré-prints e documentos institucionais não revisados, visa manter o foco em evidências robustas e amplamente aceitas pela comunidade científica, fortalecendo a validade desta síntese de evidências.

### Fontes de informação

As fontes de informação utilizadas para a busca das diretrizes clínicas foram Medline (via PubMed), Embase e Scopus, reconhecidas por indexarem periódicos científicos na área da saúde e ciências biomédicas. A busca nestas fontes objetivou aumentar a probabilidade de encontrar diretrizes elegíveis.

### Estratégia de busca

A estratégia de busca foi desenvolvida pelos autores em todas as suas fases, incluindo a elaboração, testes prévios e aplicação. A Tabela 1 descreve os detalhes da estratégia de busca utilizada neste estudo, com foco na identificação de diretrizes clínicas publicadas que abordaram o tratamento da dor crônica. A estratégia combina termos relacionados a “dor crônica”, “diretrizes” e “sistema GRADE”. A busca foi refinada pelos autores para cada base de dados, utilizando os operadores booleanos e termos específicos de cada plataforma, conforme apresentado na Tabela 1, a fim de maximizar a sensibilidade e especificidade.

**Tabela 1:** Estratégia de busca nas fontes de informação selecionadas

Fonte	Termos de busca	Estudos recuperados
Medline (**via PubMed**)	#1 “chronic pain”[Mesh] OR (chronic pain) #2 (guidelines as topic[MeSH:noexp] OR practice guidelines as topic[MeSH:noexp] OR Health Planning Guidelines[MeSH:noexp] OR practice guideline[MeSH:noexp] OR clinical protocols[MeSH:noexp] OR Consensus[MeSH:noexp] OR “Consensus Development Conference”[PTYP] OR “Consensus Development Conference, NIH”[PTYP] OR “Consensus Development Conferences as Topic”[MeSH:noexp] OR “Consensus Development Conferences, NIH as Topic”[MeSH:noexp] OR critical pathway[MeSH:noexp] OR (clinical[TIAB] AND pathway[TIAB]) OR (clinical[TIAB] AND pathways[TIAB]) OR (practice[TIAB] AND parameter[TIAB]) OR (practice[TIAB] AND parameters[TIAB]) OR algorithms[MeSH:noexp] OR care pathway[TIAB] OR care pathways[TIAB] OR guidance[TIAB] OR guideline*[TI]) #3 “GRADE system”[tiab] OR “GRADE methods”[tiab] OR “GRADE approaches”[tiab] OR “GRADE approach”[tiab] OR “Grading of Recommendations, Assessment, Development and Evaluation”[tiab] OR (“GRADE”[tiab] AND (“low”[tiab] OR “moderate”[tiab]OR “high”[tiab] OR “evidence”[tiab] OR “strong”[tiab] OR “weak”[tiab] “recommendation”[tiab])) #4 #1 AND #2 AND #3	96
Embase	#1 ‘chronic pain’/exp OR ‘chronic pain’ #2 ‘practice guideline’/exp OR ‘practice guideline’ #3 ‘GRADE approach’/exp OR ‘GRADE approach’ #4 #1 AND #2 AND #3	30
Scopus	(TITLE-ABS-KEY (“chronic pain”)) AND (TITLE-ABS-KEY(guidelines)) AND (TITLE-ABS-KEY(“GRADE system” OR “GRADE methods” OR “GRADE approaches” OR “GRADE approach” OR “Grading of Recommendations, Assessment, Development and Evaluation”))	120

### Processo de seleção

A seleção das diretrizes foi realizada em duas etapas, com auxílio de uma planilha eletrônica. Primeiramente, dois revisores independentes realizaram a triagem dos títulos e resumos dos registros identificados nas bases de dados, utilizando os critérios de elegibilidade predefinidos. As divergências entre os revisores foram resolvidas por consenso. Em seguida, os registros considerados potencialmente elegíveis após a triagem inicial tiveram o texto completo recuperado para uma avaliação detalhada por um revisor, sendo a elegibilidade averiguada por outro revisor. Um diagrama de fluxo PRISMA foi elaborado para ilustrar o processo.

### Processo de coleta de dados

Um revisor extraiu os dados das diretrizes incluídas utilizando uma planilha eletrônica padronizada e um segundo revisor revisou as informações extraídas, para fins de confirmação. As discordâncias na extração dos dados foram discutidas pelos revisores, chegando-se a um consenso.

### Lista de dados

Uma planilha foi elaborada para coletar os seguintes dados: ano de publicação; local de origem; população-alvo (tipo de dor crônica); PICS incluída; desfechos considerados; recomendação para o uso da PICS; qualidade da evidência (alta, moderada, baixa ou muito baixa) e força da recomendação (forte ou fraca).

### Avaliação do risco de viés dos estudos

Para avaliar o risco de viés das diretrizes clínicas incluídas na síntese, foi utilizado o domínio rigor metodológico da ferramenta Appraisal of Guidelines for Research and Evaluation II (AGREE II) (18). Utilizaram-se os seguintes itens: (i) Foram utilizados métodos sistemáticos para a busca de evidências? (ii) Os critérios para a seleção de evidências estão claramente descritos? (iii) Os pontos fortes e limitações do corpo de evidências estão claramente descritos? (iv) Os métodos para a formulação das recomendações estão claramente descritos? (v) Os benefícios, efeitos colaterais e riscos à saúde foram considerados na formulação das recomendações? (vi) Existe uma relação explícita entre as recomendações e as evidências que lhes dão suporte? (vii) A diretriz foi revisada externamente por experts antes da sua publicação? (viii) Um procedimento para atualização da diretriz está disponível? A aplicação da avaliação foi feita por um revisor e checada por outro. As eventuais discordâncias foram resolvidas por consenso. Ambos os revisores tinham experiência prévia na aplicação do AGREE II.

### Métodos de síntese

A análise das recomendações das diretrizes incluídas na síntese de evidências foi conduzida de forma narrativa, considerando-se a qualidade metodológica das diretrizes e a qualidade da evidência que sustenta cada recomendação. As recomendações foram agrupadas por tipo de dor crônica e modalidade de PICS, buscando-se identificar pontos de convergência e divergência entre as diferentes diretrizes.

### Avaliação da certeza

A análise dos resultados levou em consideração a qualidade metodológica das diretrizes, conferindo-se maior peso às recomendações provenientes de diretrizes com baixo risco de viés, ou seja, as mais confiáveis. Consideraram-se diretrizes com confiança alta aquelas com maior número de respostas “sim” no rigor metodológico da ferramenta AGREE II; diretrizes com confiança moderada aquelas com equilíbrio de respostas “sim”, “parcialmente” e “não”; e diretrizes com confiança baixa aquelas com a maioria dos critérios classificados como “parcialmente” e “não”.

A força das recomendações e a qualidade da evidência foram extraídas do sistema GRADE das diretrizes. No GRADE, recomendação forte indica que o painel de especialistas está confiante em que os efeitos benéficos prevalecem sobre os malefícios. Uma recomendação fraca/condicional significa que há incerteza quanto ao equilíbrio entre benefícios e riscos, de modo que a melhor decisão pode variar conforme o contexto ou os valores do paciente. A combinação das recomendações foi realizada de forma qualitativa, buscando-se um equilíbrio a partir da análise crítica do conjunto de evidências disponíveis.

## Resultados

A Figura 1 apresenta o processo de seleção de diretrizes para a síntese de evidências. A busca nas fontes de informação, em 9 de setembro de 2024, resultou em 246 registros. Após a remoção das duplicações, 201 registros foram considerados para a seleção de títulos e resumos. Desses, 35 tiveram o texto completo recuperado para avaliação. A partir da leitura dos textos completos, 19 artigos descrevendo 18 diretrizes foram incluídos na síntese por abordarem algum tipo de dor crônica e por terem alguma PICS entre suas recomendações (19-37).

**Figura 1:** Fluxo de identificação das diretrizes sobre dor crônica e uso de Práticas Integrativas e Complementares em Saúde

A Tabela 2 descreve as características das 18 diretrizes clínicas incluídas nesta síntese publicadas entre 2011 e 2024. As diretrizes são provenientes de diversos países, com destaque aos europeus e aos Estados Unidos. As populações-alvo incluíram condições como dor lombar, dor pélvica crônica, fibromialgia, síndrome do intestino irritável, pancreatite crônica e artrite reumatoide. As PICS mais recomendadas foram acupuntura, osteopatia, quiropraxia, ioga, tai chi, meditação e técnicas de relaxamento. Os desfechos avaliados incluíram a redução da intensidade da dor, melhora da funcionalidade física e emocional, qualidade de vida, sono, bem-estar psicológico e redução do uso de medicamentos.

**Tabela 2:** Caracterização das diretrizes clínicas incluídas sobre dor crônica

Autor/ano	Local de origem	População-alvo	Práticas Integrativas e Complementares em Saúde	Desfechos
Allaire 2024 (19)	Canadá	Mulheres adolescentes com dor pélvica crônica	Acupuntura	Redução da dor
Brandow 2020 (20)	Estados Unidos	Crianças e adultos com doença falciforme	Acupuntura; massagem terapêutica	Alívio da dor, redução do uso de analgésicos, bem-estar psicológico.
Broder 2022 (21)	Estados Unidos	Adultos com dor abdominal recorrente de baixo risco com entrada em emergência	Acupuntura	Redução da dor, necessidade de analgésicos de resgate e satisfação do paciente com o alívio da dor.
Busse 2023 (22)	Diversos países	Pacientes adultos com dor crônica (≥3 meses) associada a distúrbios temporomandibulares	Acupuntura	Alívio da dor, funcionamento físico, funcionamento emocional, funcionamento de papéis, funcionamento social, qualidade do sono e eventos adversos.
Coulter 2019 (23)	Estados Unidos	Pacientes com dor cervical não especificada	Osteopatia; quiropraxia	Redução da dor, redução da incapacidade e melhora na qualidade de vida.
De Andres 2016 (24)	Espanha	Mulheres com vulvodinia	Biofeedback; terapia cognitivo comportamental	Redução da dor, dispareunia, melhora da qualidade de vida.
Di Nardo 2024 (25)	Itália	Crianças e adolescentes com síndrome do intestino irritável	Ioga; técnica de relaxamento Benson	Redução da frequência e intensidade da dor abdominal, melhora da qualidade de vida, redução da gravidade dos sintomas e bem-estar psicológico.
Dominguez-Munoz 2018 (26)	Europa	Pacientes com pancreatite crônica	Acupuntura	Alívio da dor, melhora da qualidade de vida, estado nutricional, controle do diabetes, progressão da doença e complicações.
Hawk 2020 (27)	Estados Unidos	Pacientes com dor musculoesquelética crônica	Acupuntura; osteopatia; quiropraxia; ioga; meditação mindfulness; terapia cognitivo-comportamental	Controle da dor, função, qualidade de vida, saúde mental, comportamentos relacionados à dor, uso de medicamentos, satisfação do paciente, custos e eventos adversos.
Krenn 2020 (28)	Áustria	Adultos com lombalgia inespecífica	Acupuntura; medicina tradicional chinesa; fitoterapia; osteopatia; quiropraxia; ioga; meditação; musicoterapia	Redução da dor, melhora da funcionalidade, redução do uso de medicamentos.
Larun 2011 (29)	Noruega	Pacientes com síndrome da fadiga crônica	Qigong	Níveis de fadiga, dor e qualidade de vida relacionada à saúde.
Lohr 2017 (30)	Europa	Pacientes com pancreatite crônica	Acupuntura	Alívio da dor, qualidade de vida, morbidade, mortalidade e custos do tratamento.
Moisset 2020 (31, 32)	França	Pacientes com dor neuropática crônica, incluindo adultos e crianças	Acupuntura auricular; aromaterapia; massagem com aromaterapia; laser de baixa intensidade	Intensidade da dor, sintomas e sinais neuropáticos, sono, qualidade de vida, comorbidades, impressão clínica global de mudança e eventos adversos.
Pangarkar 2019 (33)	Estados Unidos	Pacientes com dor lombar (incluindo veteranos e militares)	Acupuntura; osteopatia; quiropraxia; ioga; meditação mindfulness; terapia cognitivo-comportamental	Dor, função, qualidade de vida, saúde mental, comportamentos relacionados à dor, uso de medicamentos, satisfação do paciente, custos e eventos adversos.
Qaseem 2017 (34)	Estados Unidos	Adultos com lombalgia aguda, subaguda ou crônica	Acupuntura; osteopatia; quiropraxia; massagem; tai chi; ioga; mindfulness	Redução da dor, melhora da função, melhora da qualidade de vida relacionada à saúde, redução da incapacidade laboral, retorno ao trabalho, melhora global, número de episódios de dor lombar ou tempo entre os episódios, satisfação do paciente.
Sauvan 2018 (35)	França	Mulheres com endometriose sem infertilidade	Ioga; osteopatia; acupuntura	Melhora da qualidade de vida e redução da dor.
Van Doorn 2023 (36)	Europa	Pacientes com síndrome de Guillain-Barré	Ioga; meditação	Qualidade do sono, dor, atividades diárias e qualidade de vida.
Zhang 2024 (37)	Diversos países	Pessoas com artrite reumatoide ativa	Extrato de *Tripterygium wilfordii* *Hook F*	Resposta clínica (ACR20/50/70^a^, DAS28^b^, EULAR^c^, cDAI^d^), dor avaliada pelo paciente (VAS^e^), qualidade de vida (SF-36^f^, HAQ^g^), progressão radiográfica, segurança e custo.

^a^ American College of Rheumatology Response Criteria 20%, 50% e 70% de melhora; ^b^ Disease Activity Score in 28 Joints; ^c^ European League Against Rheumatism; ^d^ Clinical Disease Activity Index; ^e^ Visual Analog Scale; ^f^ Short Form 36; ^g^ Health Assessment Questionnaire.

A Tabela 3 apresenta a avaliação da qualidade metodológica das diretrizes clínicas incluídas na síntese. Oito diretrizes demonstraram qualidade metodológica alta, indicando maior rigor metodológico e transparência no processo de desenvolvimento. As demais diretrizes apresentaram falhas em critérios como a descrição detalhada dos métodos de busca e seleção de evidências, a análise crítica do corpo de evidências e a explicitação da relação entre as recomendações e as evidências, resultando em classificações de qualidade moderada ou baixa.

**Tabela 3:** Avaliação crítica das diretrizes clínicas incluídas sobre dor crônica

Autor/ano	1^a^	2^b^	3^c^	4^d^	5^e^	6^f^	7^g^	8^h^	Confiança da diretriz
Allaire 2024 (19)	S^i^	P^j^	S	S	S	S	S	S	Moderada
Brandow 2020 (20)	S	P	S	S	S	P	S	S	Moderada
Broder 2022 (21)	S	S	S	S	S	P	S	S	Moderada
Busse 2023 (22)	S	S	S	S	S	S	N^l^	N	Moderada
Coulter 2019 (23)	S	S	P	N	S	S	S	N	Baixa
De Andres 2016 (24)	S	P	S	N	P	S	N	N	Baixa
Di Nardo 2024 (25)	S	S	S	S	S	S	S	N	Alta
Dominguez-Munoz 2018 (26)	P	N	P	P	S	P	S	N	Baixa
Hawk 2020 (27)	S	S	S	S	S	S	S	N	Alta
Krenn, 2020 (28)	S	S	S	N	P	S	N	P	Baixa
Larun 2011 (29)	S	S	S	N	S	P	N	N	Baixa
Lohr 2017 (30)	S	S	S	S	S	S	S	S	Alta
Moisset 2020 (31, 32)	S	S	S	S	S	S	S	N	Alta
Pangarkar 2019 (33)	S	S	S	S	S	S	S	N	Alta
Qaseem 2017 (34)	S	S	S	S	S	S	S	P	Alta
Sauvan 2018 (35)	S	P	S	S	S	S	S	N	Moderada
Van Doorn 2023 (36)	S	S	S	S	S	S	S	S	Alta
Zhang 2024 (37)	S	S	S	S	S	S	S	N	Alta

^a^1. Foram utilizados métodos sistemáticos para a busca de evidências?; ^b^ 2. Os critérios para a seleção de evidências estão claramente descritos?; ^c^ 3. Os pontos fortes e limitações do corpo de evidências estão claramente descritos?; ^d^ 4. Os métodos para a formulação das recomendações estão claramente descritos?; ^e^ 5. Os benefícios, efeitos colaterais e riscos à saúde foram considerados na formulação das recomendações?; ^f^ 6. Existe uma relação explícita entre as recomendações e as evidências que lhes dão suporte?; ^g^ 7. A diretriz foi revisada externamente por experts antes da sua publicação?; ^h^ 8. Um procedimento para atualização da diretriz está disponível?; ^i^ Sim; ^j^ Parcialmente; ^l^ Não.

A Tabela 4 sintetiza as recomendações de uso de PICS para diferentes tipos de dor crônica. As recomendações variam conforme a condição específica e a modalidade de PICS. A maioria das diretrizes sugere seu uso como tratamento complementar, enfatizando a importância de considerar as preferências do paciente. A qualidade da evidência que sustenta essas recomendações é predominantemente baixa a moderada, resultando em recomendações majoritariamente fracas. A confiança nas diretrizes também varia, de baixa a alta. As diretrizes com maior grau de confiança recomendam algumas PICS para o manejo da dor crônica com ressalvas. Acupuntura, osteopatia, quiropraxia, ioga e tai chi apresentam recomendação forte, sugerindo um maior respaldo sobre seu desempenho para dor lombar. Ressalta-se que, mesmo nas diretrizes com maior confiança, as recomendações são fracas, refletindo as limitações da evidência disponível.

**Tabela 4:** Síntese das recomendações de uso das Práticas Integrativas e Complementares em Saúde em diretrizes clínicas sobre dor crônica

População-alvo	Recomendação	Qualidade da evidência	Força da recomendação	Confiança da diretriz
**Sistema musculoesquelético**				
Distúrbios temporomandibulares	Acupuntura pode ser considerada (22).	Baixa	Fraca	Moderada
Dor cervical	Osteopatia e quiropraxia podem ser consideradas opções viáveis para o tratamento da dor e redução da incapacidade (23).	Moderada	Fraca	Baixa
	A acupuntura pode ser benéfica como parte de um plano de tratamento multimodal, especialmente no início, para auxiliar o paciente a se tornar ou permanecer ativo (27).	Moderada	Fraca	Alta
Dor lombar aguda ou subaguda	Acupuntura, osteopatia e quiropraxia podem ser consideradas (34).	Baixa	Forte	Alta
Dor lombar crônica	A acupuntura pode ser oferecida (28,33,34). Ioga e tai chi podem ser oferecidos como parte de um programa de exercícios (28,33,34).	Baixa	Forte	Alta
Dor pélvica crônica	Massagem pode ser considerada (34).	Moderada	Fraca	Alta
	Acupuntura pode ser considerada uma modalidade complementar (19,35).	Baixa	Fraca	Moderada
Fibromialgia	A acupuntura pode ser benéfica (27).	Moderada	Fraca	Alta
Osteoartrite	A acupuntura, usando “alta dose” (maior frequência de tratamento, pelo menos três vezes por semana), pode ser benéfica (27).	Moderada	Fraca	Alta
**Condições sistêmicas e inflamatórias**				
Artrite reumatoide	A monoterapia com extrato de *Tripterygium wilfordii Hook F* ou sua associação com metotrexato podem ser uma opção de primeira linha (37).	Moderada	Fraca	Alta
Doença falciforme	Massagem terapêutica e acupuntura devem ser sugeridas conforme disponíveis, toleradas e condicionadas à preferência e resposta individual do paciente (20). Essas abordagens devem ser fornecidas no contexto de um plano abrangente de gestão da doença e da dor (20).	Muito baixa	Fraca	Moderada
Dor neuropática crônica	A terapia cognitivo-comportamental e a terapia de mindfulness podem ser recomendadas como terapia de segunda linha, em adição a outras terapias (31, 32). A hipnose, a reflexoterapia e a acupuntura podem ser eficazes, mas pesquisas adicionais são necessárias (31, 32).	Moderada	Fraca	Alta
Endometriose	Ioga, osteopatia e acupuntura podem ser propostos como parte da abordagem não medicamentosa para melhorar a qualidade de vida (35).	Baixa	Fraca	Moderada
Pancreatite crônica	Acupuntura pode ser considerada em casos de dor (26,30).	Moderada	Fraca	Alta
Síndrome do intestino irritável	Ioga e relaxamento podem ser considerados (25).	Muito baixa	Fraca	Alta
Síndrome da fadiga crônica	Exercício terapêutico pode ser recomendado, desde que o programa seja adaptado à natureza da doença (29).	Moderada	Fraca	Baixa
Síndrome de Guillain-Barré	Ioga e meditação podem ser consideradas para o tratamento de dor e fadiga, mas pesquisas adicionais são necessárias (36).	Muito baixa	Fraca	Alta
**Outras condições e sintomas**				
Dor abdominal	Acupuntura pode ser considerada (21).	Muito baixa	Fraca	Moderada
Vulvodínia	O biofeedback pode ser considerado como parte de uma abordagem multidisciplinar, especialmente em casos de disfunção do assoalho pélvico (24). A terapia cognitivo-comportamental é recomendada como parte de uma abordagem multidisciplinar, juntamente com psicoterapia de apoio (24).	Moderada	Fraca	Baixa

## Discussão

As diretrizes clínicas que contemplam o uso de PICS para dor crônica revelam um cenário de interesse crescente, mas com base de evidências ainda limitada. Embora muitas diretrizes, especialmente de origem europeia e americana, abordem o tema para diferentes condições dolorosas, a qualidade metodológica dessas diretrizes é heterogênea, com muitas apresentando fragilidades na transparência e rigor do processo de desenvolvimento. Com exceção do uso de acupuntura, osteopatia, quiropraxia, ioga e tai chi para dor lombar, as recomendações são fracas, refletindo evidências de qualidade predominantemente baixa a moderada. Isso sugere que, embora haja indícios do potencial benefício das PICS no manejo da dor crônica, mais pesquisas robustas são necessárias para consolidar essas práticas e definir seu papel de forma mais precisa na prática clínica. A ênfase na individualização do tratamento e na consideração das preferências do paciente reforça a necessidade de uma abordagem cautelosa e integrada, com base em evidências e na comunicação compartilhada.

A síntese de evidências apresenta limitações. A heterogeneidade das diretrizes clínicas incluídas, tanto em termos de escopo (diferentes tipos de dor crônica) quanto de qualidade metodológica, dificulta a generalização das conclusões (38). A predominância de evidências de baixa a moderada qualidade, que sustentam as recomendações, introduz imprecisão e limita a força das conclusões (39). Potenciais vieses de publicação, com estudos negativos ou inconclusivos tendo menor probabilidade de serem publicados, podem ter influenciado os resultados (40). A busca por diretrizes, mesmo realizada em bases de dados relevantes, não capturou todas as diretrizes existentes, especialmente as não indexadas. 

PICS tem potencial para o manejo da dor crônica, oferecendo uma opção custo-efetiva e com perfil de segurança favorável em comparação a tratamentos convencionais. Modalidades como acupuntura e práticas mente-corpo abordam a complexidade da dor crônica, minimizando os efeitos adversos frequentemente associados aos medicamentos (41). A integração criteriosa das PICS à medicina convencional pode otimizar recursos, reduzir efeitos colaterais e aumentar a satisfação do paciente (42). Esta síntese aponta as PICS mais promissoras para cada tipo de dor crônica, as quais podem ser integradas aos tratamentos convencionais disponíveis no SUS.

A acupuntura, por exemplo, já está incluída na Política Nacional de Práticas Integrativas e Complementares no SUS e demonstrou benefícios em diretrizes internacionais, representando uma oportunidade para ampliar o acesso e reduzir iniquidades (43). A capacitação de profissionais do SUS em PICS, como ioga e meditação, com potencial para serem oferecidas em diversos níveis de atenção, pode fortalecer a autonomia do paciente e a promoção da saúde. A inclusão criteriosa de PICS amplia as opções terapêuticas, promove o cuidado integral e fortalece o SUS, garantindo acesso equitativo a práticas seguras e eficazes.

A Secretaria Municipal da Saúde de São Paulo padronizou o manejo da dor crônica em centros de referência do município (44). Embora o foco principal seja o tratamento medicamentoso e intervenções convencionais, se reconhece a importância das PICS como parte de uma abordagem multidisciplinar. Entre as PICS mencionadas, estão acupuntura, auriculoterapia, moxabustão, reflexologia, tai chi, ventosaterapia, fitoterapia, meditação e ioga. O protocolo clínico cita revisões sistemáticas que demonstram a efetividade dessas práticas para diferentes tipos de dor crônica, incluindo dor lombar, cervical, musculoesquelética e dor oncológica. A inclusão das PICS no protocolo reforça a tendência de integrar essas abordagens ao SUS, visando a um manejo mais abrangente e individualizado da dor crônica.

O Protocolo Clínico e Diretrizes Terapêuticas da Dor Crônica (PCDT), publicado em agosto de 2024, reconhece a importância das PICS. Entre as PICS destacadas, estão a acupuntura e a auriculoterapia, que têm demonstrado eficácia na redução da dor em adultos e idosos (45). Essas práticas são recomendadas como parte de uma abordagem terapêutica multidisciplinar, visando melhorar a qualidade de vida dos pacientes. O PCDT enfatiza a necessidade de profissionais capacitados para a aplicação dessas técnicas, garantindo a segurança e a efetividade do tratamento. Também é reforçada a importância de sevconsiderarem as preferências e particularidades de cada paciente na elaboração do plano terapêutico.

Em conclusão, esta síntese de evidências demonstra que, embora o uso de PICS para dor crônica seja promissor e crescente, a qualidade da evidência ainda é um fator limitante para a formulação de recomendações robustas. As recomendações se fortalecem ao serem considerados o custo-efetividade e a segurança destas práticas. Acupuntura, osteopatia, quiropraxia e ioga emergem como práticas com maior suporte para dor lombar. Profissionais de saúde devem integrar essas evidências com sua experiência clínica, considerando as preferências e necessidades individuais de cada paciente, na decisão sobre o uso de PICS. A ampliação do acesso às PICS no SUS, a capacitação profissional e o investimento em pesquisas no contexto brasileiro são essenciais para promover o uso seguro e efetivo dessas práticas, contribuindo para o cuidado integral e para a sustentabilidade do sistema.

## Data Availability

Não houve criação de banco de dados e/ou códigos de análise nesta pesquisa, além das ilustrações já disponíveis no manuscrito.
